# The regulatory roles of motile cilia in CSF circulation and hydrocephalus

**DOI:** 10.1186/s12987-021-00265-0

**Published:** 2021-07-07

**Authors:** Vijay Kumar, Zobia Umair, Shiv Kumar, Ravi Shankar Goutam, Soochul Park, Jaebong Kim

**Affiliations:** 1 Department of Biochemistry, Institute of Cell Differentiation and Aging, College of Medicine, Hallym University, Gangwon-Do, Chuncheon, 24252 Republic of Korea; 2 Department of Molecular Medicine, School of Medicine, Gachon University, Incheon, 21999 Republic of Korea; 3 School of Psychology and Neuroscience, University of St. Andrews, St. Mary’s Quad, South Street. St. Andrews, Fife, KY16 9JP UK; 4 Department of Biological Sciences, Sookmyung Women’s University, Seoul, 04310 Republic of Korea

**Keywords:** Cerebrospinal fluid, Cilia, Ependymal cells, Brain development, Ciliary motility, Brain ventricular system, Hydrocephalus, Ciliopathies

## Abstract

**Background:**

Cerebrospinal fluid (CSF) is an ultra-filtrated colorless brain fluid that circulates within brain spaces like the ventricular cavities, subarachnoid space, and the spine. Its continuous flow serves many primary functions, including nourishment, brain protection, and waste removal.

**Main body:**

The abnormal accumulation of CSF in brain cavities triggers severe hydrocephalus. Accumulating evidence had indicated that synchronized beats of motile cilia (cilia from multiciliated cells or the ependymal lining in brain ventricles) provide forceful pressure to generate and restrain CSF flow and maintain overall CSF circulation within brain spaces. In humans, the disorders caused by defective primary and/or motile cilia are generally referred to as ciliopathies. The key role of CSF circulation in brain development and its functioning has not been fully elucidated.

**Conclusions:**

In this review, we briefly discuss the underlying role of motile cilia in CSF circulation and hydrocephalus. We have reviewed cilia and ciliated cells in the brain and the existing evidence for the regulatory role of functional cilia in CSF circulation in the brain. We further discuss the findings obtained for defective cilia and their potential involvement in hydrocephalus. Furthermore, this review will reinforce the idea of motile cilia as master regulators of CSF movements, brain development, and neuronal diseases.

## Background

Cerebrospinal fluid (CSF), a colorless liquid, is essential for brain homeostasis and functioning. Growing evidence has revealed the various crucial roles of CSF throughout the lifespan of vertebrates. During the early development of vertebrates, the central nervous system (CNS) develops from the neural tube (a hollow pipe-like structure), which is filled with embryonic CSF (eCSF). Studies have documented that eCSF contains several types of diffusible neurotrophic agents, growth factors, and other cytokines, which directly regulate neural differentiation, proliferation, and development, and other neural functions [1–4]. Since CSF circulation requires an external forceful and precise pressure, motile cilia play an essential role in propelling the CSF within the brain and spinal cavities. Collectively, CSF dynamics are heavily dependent on the condition of motile cilia. Thus, defects in cilia development and cilia motility disrupt synchronization of and restrain CSF circulation, causing CSF accumulation in the ventricles. Furthermore, these ciliary defects are widely associated with several human diseases, commonly known as ciliopathies [5, 6]. Ciliopathies constitute a large group of human disorders generally caused by mutations in genes encoding the proteins involved in multiciliated cell (MCC) differentiation and proliferation or cilia formation. To date, more than 200 candidate genes have been recognized to be associated with ciliopathies and can be categorized by their involvement in ciliogenesis/signaling, structure, and the functions of motile or non-motile cilia. The common ciliopathies associated with motile cilia include hydrocephalus (brain), situs inversus (left–right asymmetry) chronic respiratory problems (respiratory system), and infertility (reproductive system) [7, 8]. In the brain, cilia-driven CSF flow is significantly involved in brain functioning, which has recently been reviewed by Fame and Lehtinen [9]. Additionally, CSF may be involved in the processes of early body axis patterning and left/right asymmetry, which are most notably linked to early brain development [10, 11].

The healthy brain contains three integrated components that collectively regulate CSF dynamics (or the CSF cycle): the amount of CSF produced, the amount of CSF in circulation, and the amount of CSF available for absorption. These three components typically remain in equilibrium. Disruption in any of these components, e.g., by overproduction or defective circulation, causes excessive accumulation of CSF in the brain and reduces the amount of CSF available for reabsorption, resulting in ventriculomegaly. Ventriculomegaly is one of earliest signs of hydrocephalus and other severe brain pathologies. Impairments in the functioning of motile cilia accentuate the hydrocephalus pathologies attributable to abnormal CSF circulation [12–14]. Over the last few decades, several studies have attempted to elucidate the relationship between motile cilia and hydrocephalus. Some evidence highlights motile cilia as a unique machinery of ciliated cells that not only propels the CSF but also leads the embryonic development of vertebrates, including left–right body asymmetry, brain development, and brain functioning. In this review, we briefly discuss the critical functional roles of motile cilia in CSF circulation and how dysfunctional and defective ciliary movements disturb the overall CSF dynamics. Finally, we present evidence from experimental animal research indicating that a functional mutation in proteins associated with ciliogenesis and MCC differentiation results in development of dysfunctional cilia, which subsequently results in poor CSF circulation, leading to severe brain disorders, including hydrocephalus.

## CSF secretion and absorption

**Cerebrospinal fluid (CSF)**, the colorless liquid circulating within spaces surrounded by the brain and the spinal cord, has a long history of discovery that has been well outlined by Hajdu [15]. The **choroid plexus (CP)**, a complex vascular structure of epithelial and endothelial cells within the ventricular system of the vertebrate brain, is known to be a significant source of CSF secretion [16–18]. The endothelial layer of capillaries in the CP is permeable to solutes, and solutes freely enter the stromal space. In contrast, the epithelial cell membrane of the CP exposes several transporters, channels, and aquaporins at both basal and apical surfaces and functions as a filter for ions and cations by regulating diffusion and facilitating the selective transport of some solutes. In dogs, the extra-ventricular space was shown to produce approximately 58.5% of the total CSF [18], whereas the extra-choroid proportion in rabbits accounted for 33% of overall CSF [19]. These observations indicate that a substantial amount of CSF is produced from the extra-choroidal space. In humans, CSF volume has been measured to be between 400 to 600 mL per day, and it can be renewed up to 4 to 5 times in one day, with approximately 150 mL of CSF circulating in the brain and related organs, approximately 125 mL in the subarachnoid space, and approximately 25 mL in the ventricles of the brain [20].

The starting ventricular system CSF enters the subarachnoid space at the base of the brain laterally at the foramen of Lushka and medially at the foramen of Magendie [21]. The CSF moves along the brain axis from the CP to the site of absorption and continuously circulates through the superior sagittal sinus, intracranial venous sinuses, and spaces around the spinal cord [22]. However, the mechanism of CSF absorption is a complicated process, and several studies have indicated that CSF absorption occurs at different locations. The arachnoid villi are endothelium-lined protrusions that may show several morphologies and are present in the meningeal sheath of spinal nerve roots and other locations within the brain, sites primarily known to show CSF absorption [22, 23]. In addition to the arachnoid villi (also known as arachnoid granulations), extracranial lymphatic vessels (40% to 48%) substantially contribute to overall glymphatic CSF circulation in adult sheep [24]. However, recent discoveries indicate that the glymphatic system (glia plus lymphatic) and meningeal lymphatic system actively contribute to overall brain fluid drainage, which has been reviewed elsewhere [25–30]. Although, these studies demonstrate the possible mechanism of CSF reabsorption, the exact mechanism remains to be fully elucidated.

## Functions of the CSF

### In brain functioning

The continuous circulation of CSF assists in the exchange of essential biomolecules, providing fresh nutrients as well as removing waste products generated by the brain [31]. The composition and acid–base balance of the CSF is tightly regulated and directly manipulated through the epithelial cells of the CP. These cells carry a large number of active transporters, passive transporters, and other types of channels (on the surface, luminal, and basolateral membrane) that are switched on or off [32] and can directly facilitate maintenance of the composition of the brain fluid by the influx and outflux of several types of ions, biomolecules, proteins, and other components [33]. In general, CSF facilitates mechanical support and maintenance of homeostasis, and its other crucial functions may include the distribution of neurotransmitters and neuroactive hormones to remote sites of the CNS [32].

### In brain development

The CSF is essential for brain functioning in adult animals, and a number of studies in the past decades have strongly supported the crucial role of CSF in early developmental processes, including those involving the brain and body axis. The earliest form of CSF, known as eCSF, is found in the neural tube during the embryonic and fetal development of vertebrates [34]. Generally, eCSF is a protein-rich fluid and differs from adult CSF mainly in terms of composition [35, 36]. eCSF regulates different aspects of early brain development, while fluid pressure dramatically influences brain growth and morphogenesis. In vitro experiments indicate that the presence of eCSF induces neuroepithelial cell survival, proliferation, and differentiation [2, 37]. In rat and chick embryos, the accumulation of eCSF inside the ventricular system creates hydrostatic pressure, which significantly affects expansion and morphogenesis. These studies indicated that eCSF contains active molecules or growth factors and can therefore directly regulate neuroepithelial cell behavior as well as proliferation. The evidence shows that eCSF contains several active growth factors, including fibroblast growth factor 2 (Fgf2), insulin growth factor 2 (Igf2), and other neurogenesis modulators, which play critical roles and drive neurogenesis [38, 39], and lipoproteins that can interact with the neuroepithelium and may trigger signal transduction through several growth factors [40]. Interestingly, comparative analysis of eCSF between humans and rats has revealed the presence of common neurogenic factors, such as amyloid beta A4 protein precursor and tenascin, which are known to play an active role in brain growth and development [1]. However, we recommend readers visit a recent review on the role of the CSF in brain development [9]. In addition, ependymal flow is essential for neuroblast migration, which indicates CSF has functions beyond the provision of nutrients [41]. These studies suggest that eCSF contains all of the factors necessary to modulate neural cell proliferation and differentiation [1–4].

## Ciliated cells and their distribution in the brain

During the early development of the human brain, neural progenitors (neuroepithelial or radial glial cells) undergo differentiation, proliferation, and migration to form a mature complex neural network [42, 43]. The functional cytoarchitecture of the adult human brain, from the fetal to the postnatal phase, requires a lot of time for development. The size of the brain and its cellular composition varies across mammalian species, and these differences might result in different orders of species and evolution. This variance also influences the differences in cognitive abilities across different species of vertebrates [44]. However, cilia represent a common organelle found in the vertebrate brain, including in neurons and glial cells. Mammalian cells contain two types of cilia: primary (non-motile or immotile) cilia and motile cilia [45]. The majority of animal tissues, including the epithelium, nerve, and connective tissue, carry primary cilia [46]. The major roles of primary cilia include sensory perception, signal transduction, cell cycle progression, and brain development [47]. In contrast, motile cilia play important roles in fluid circulation or movements and are widely distributed across the plant and animal kingdoms [48]. In the animal brain, motile cilia are a hallmark of ependymal epithelial cells, which are differentiated from glial cells [49]. The ependymal cells (ECs) can be further classified into three groups on the basis of the cilia exposed on their apical surface. The first type of ECs (E1) are multi-ciliated with an abundant number (20 to 100 cilia/cell) of motile cilia. The second group includes bi-ciliated ECs (E2) with one or two motile cilia extending from the apical surface. These cells are generally referred to as α-tanycytes and are abundantly found in the third ventricle and extend into the aqueduct and fourth ventricle [50, 51]. The third group contains uni-ciliated ECs (E3) with one non-motile cilia; these cells are known as β-tanycytes and are found in abundance in the floor of the third ventricle [50, 51]. These ECs (neuroglia) form the epithelial lining of the brain’s ventricle system, central canal, spinal cord, and CP. The innermost layer of the ventricles is formed by ECs with a ciliated apical surface exposed to the ventricular cavity, wherein ciliary beating plays a key role in propelling CSF throughout the ventricles [52, 53]. A magnetic resonance imaging (MRI) study investigated the nature of fluid flow and demonstrated that ependymal cilia generated flow dominates along the walls of the lateral ventricles rather than in the middle of the ventricles [54]. However, several studies suggested that the number of ECs in the third ventricle can vary, as can the length of the cilia on the apical surface [55]. The diversity among the cilia number and length might be linked to the diversity of ECs (including special types of ECs, i.e., α-tanycytes and β-tanycytes) [51, 56].

## Ciliary structure and ciliogenesis

**Fig. 1:**
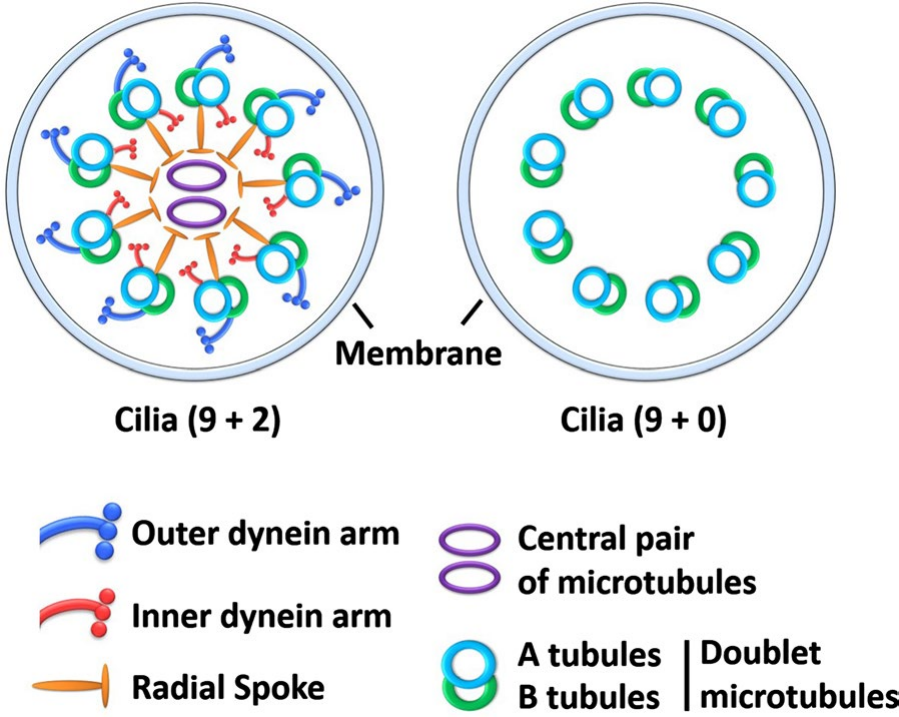
Structural differences between cross-section of motile (9 + 2) and primary (9 + 0) cilia

The cilia are highly conserved and widely dispersed throughout the eukaryotic kingdom. Over the last few decades, cilia have emerged as essential organelles involved in various physiological and developmental processes [56]. Typically, cilia are divided into primary and motile cilia. These two types of cilia show distinct structural and functional characteristics. The immotile, simple hair-like extensions from the cell surface are mainly involved in sensing the several extracellular morphogens (signals) and normally present on nearly all human cells [57–59]. In contrast, motile cilia are primarily present on the epithelial surfaces of the oviduct, trachea, and ependymal lining of the brain ventricles, and their synchronized beats generate fluid movement [60–64]. Cilia have a complex structure, and their cytoskeleton is primarily composed of microtubule-based structures called axonemes and the ciliary membrane. The axoneme itself contains a ring of nine microtubule doublets (MTD) surrounded by the ciliary membrane, and in the center of this ring is a singlet pair of microtubules that is absent in primary cilia (Fig. 1). This central pair of microtubules drives the motility of motile cilia and is the primary structural difference between motile and immotile cilia, which are defined as (9 + 2) and (9 + 0), respectively [65]. Structurally, a single MTD contains two cylindrical microtubules, of which the A-tubule has a complete cylindrical structure and the B-tubule has an incomplete cylindrical structure and is attached to the A-tubule (Fig. 1). The MTD itself is directly connected with dyneins/dynein regulatory complex, which is located between the outer dynein arm (ODA) and the inner dynein arm and is connected with the CP via the radial spoke protein [66, 67]. The axoneme connects to or is extended from the distal end of the basal body (BB), the region known as the transition zone [68].

The process of cilia formation and maturation is known as ciliogenesis. It is based on two types of mechanisms, i.e., compartmentalized and cytosolic ciliogenesis [66]. Several members of the *fox* and *rfx* families have been identified in vertebrates to play essential roles in ciliogenesis (reviewed by Choksi et. al.) [69]. However, transcriptional regulation of motile cilia biogenesis or multiciliogenesis is known to be regulated by some key regulators. For example, GemC1/Lynkeas activates the *mcidas*, *myb,* and *foxj1* genes encoding transcription factors (TFs) and acts as a master regulator for early MCC differentiation and motile ciliogenesis [69–73]. GemC1 physically interacts with E2F5 (TF) and p73/TP73 (tumor protein); this heterotrimeric complex is required for the activation of *p73* and *foxj1,* and to drive the multiciliogenic process in vertebrates [70, 72]. Additionally, multicilin acts via Notch signaling, and its knockdown completely blocks MCC differentiation and *foxj1* transcriptional activation in Xenopus [74]. However, three master regulators, Foxj1, Mcidas, and Rfx3, are known to control the biogenesis of motile cilia, but the precise transcriptional regulation mechanism remains to be elucidated [69, 70, 73–75].

## Ciliary beats control CSF circulation

**Fig. 2:**
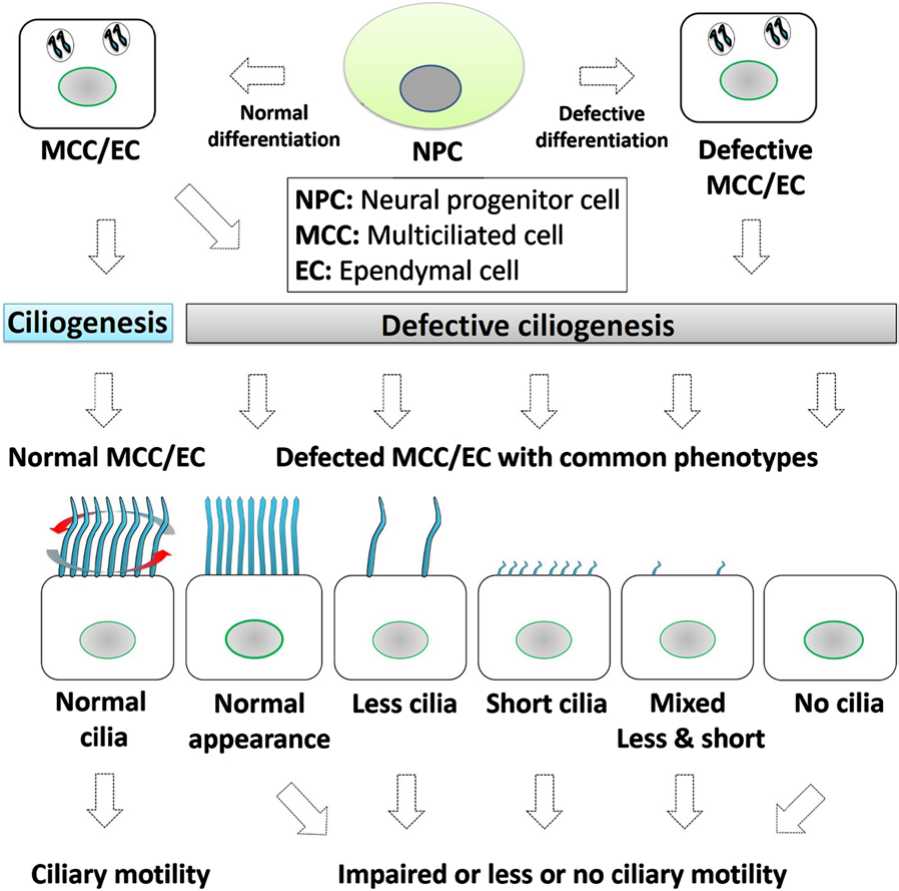
Defective ciliary phenotypes. The mutations within several structural or functional proteins trigger defective phenotype formation

The flow of the CSF is essential to supply fundamental nutrients and other biomolecules for normal brain function, which is maintained by continuous distribution of several growth/signaling factors, balancing of ion concentrations by passive diffusion to support brain homeostasis, and the removal of several metabolic byproducts produced by the brain [76]. The unidirectional flow of the CSF has been reported to be directly dependent on motile cilia, with the coordinated beats of cilia in a defined orientation generating forceful CSF movement. In this section, we briefly summarize the evidence from human and experimental animal models that strongly indicates that synchronized ciliary beats control CSF circulation, and that disrupted ciliary beating severely impairs CSF movement. Several previous loss-of-function studies of ependymal/cilia-specific genes demonstrated a partial or severe reduction in ciliary length and numbers, which collectively impaired ciliary motility and CSF flow and promoted abnormal brain development/functions, such as hydrocephalus and other ciliopathies. Based on previous reports, there are several types of defective ciliary phenotypes commonly observed in different models. Therefore, we have grouped them into five different types; in type 1, the cilia show a normal appearance, but their movement is partially or severely compromised, whereas in type 2, the number of cilia is massively reduced. In type 3, the defective cilia often show a markedly reduced length. In type 4, the defective cilia show a mixed phenotype consisting of few and short cilia, and this mixed phenotype accounts for most of the defective cilia. In type 5, the cilia are lost, and no cilia are present on the surface of MCCs/ECs (Fig. 2 and Table 1). These different phenotypes have one common feature, i.e., partially or wholly disrupted ciliary motility, which further impairs CSF circulation and leads to neurological disorders.

**Table 1:** List of genes that showed loss-of-function with different sets of ciliary phenotypes and hydrocephalus

Gene name	Average ciliary phenotype	Species	Defected ciliary motility	Hydrocephalus	References
Lrrc6	Normal	Mice	Yes	Yes	[121]
Hydin	Normal, stall	Mice	Yes	Yes	[122]
Dnaaf1	Normal	Mice	Yes	Yes	[123]
Lrrc48	Normal	Mice	Yes	Yes	[123]
CFAP54	Normal	Mice	Yes	Yes	[124]
Mpdz	Normal	Mice	Yes	Yes	[125]
Mdnah5	Normal	Mice	Yes	Yes	[126]
Zmynd10	Normal	Mice	Yes	Yes	[127]
Rsph9	Ciliary membrane inclusions	Mice	Yes	Yes	[115]
Ulk4	Lesser	Mice	Yes	Yes	[87]
*Ccdc39*	Shorter	Mice	Yes	Yes	[113]
*Jhy*	Shorter	Mice	Yes	Yes	[128]
Snx27	Shorter	Mice	Yes	Yes	[86]
*Ccdc40*	Shorter	Mice/zebrafish	Yes	Yes	[129]
Dyx1c1	Shorter	zebrafish	Yes	Yes	[130]
Celsr 1 and celsr 2	Shorter, and Lesser	Mice	Yes	Yes	[83]
Dvls (Dvl1, 2 and 3)	Shorter, and Lesser	Mice	Yes	Yes	[114]
MT1-MMP	Shorter, Lesser and Disorganized	Mice	Yes	yes	[131]
Stumpy	Absent, severely deformed	Mice	Yes	Yes	[85]
Pcdp1		Mice	Yes	Yes	[132]
CFAP43		Mice	Yes	Yes	[133]
*Ccdc151*		Mice	Yes	Yes	[134]
*Foxj1*	Shorter, and Lesser	Xenopus	Yes	Yes	(97)

Mice provide an excellent platform for studying the novel molecular mechanisms underlying gradational ciliogenesis and their use permits genetic manipulations that can improve our understanding of these mechanisms. The knockdown/mutation of different groups of genes, including those involved in signal transduction, structure, and ciliary elongation/maturation, and the modification or deletion of these genes has been shown to cause several ciliopathies that have been previously reviewed [77]. The planar cell polarity (PCP) signaling pathway plays diverse and essential roles in embryonic development, differentiation, and axis patterning in vertebrates. The key PCP effectors, including *vangl2*, *frizzled, dsh, intu,* and *fuz*, markedly control ciliogenesis and other cellular functions, and deletions or mutations of PCP target genes severely affect the molecular mechanisms underlying ciliary development and generate different types of ciliary phenotypes (as classified above) [78–80]. The Cadherin EGF Laminin Seven Pass Receptor (*Celsr)* 1‒3 genes play multiple vital functions in the CNS, with *Celsr1* being mainly involved in PCP, neural differentiation, maturation, and neural tube closure; *Celsr1*-deficient mice show defective ciliary coordination despite presenting with normal ciliogenesis [81, 82]. Moreover, *Celsr2* and *Celsr3* are involved in the functional and developmental organization of cilia [81, 83] and are highly expressed in the ependymal layer of the brain ventricles. In double-mutant mice, ependymal ciliogenesis is severely compromised, resulting in the absence of cilia or a severe reduction in the number of cilia, both of which cause defective CSF dynamics and hydrocephalus [83, 84]. A similar ciliary phenotype was observed in the *stumpy* knockdown mouse, where conditional loss of *stumpy* resulted in the absence or impairment of cilia in the brain, thereby interrupting CSF circulation and causing hydrocephalus and polycystic kidney disease [85].

Similarly, the deletion of *Snx27*, which was previously known to play an essential role in normal brain function by suppression of y-secretase-dependent amyloid precursor protein, as well in Notch cleavage, triggers the hyperactivation of Notch and produces a disorganized ependymal cell lining of the ventricles, which may result in defective ependymal differentiation. The delicate balancing of the Notch gradient and modification of Notch signaling through *Snx27* in a spatiotemporal manner appears to be critical for ependymal cell differentiation and ciliogenesis [86]. Consistent with previous reports, deletion of Unc51-like kinase 4 (*Ulk4*) triggers a similar phenotype, in which the number of ciliary bundles is dramatically reduced and disorganized, although ciliary length is not substantially affected. This ablation of ciliary organization and coordination inhibits directional beating and impairs CSF flow [87]. Over the last few decades, lower vertebrates such as *Xenopus* and zebrafish have emerged as suitable animal models to study several human genetic diseases, including ciliopathies [88–91]. These models offer many advantages over other animal models, such as easy handling and maintenance, a short developmental period, and easy genetic manipulation, making them ideal for elucidating the developmental mechanisms underlying human congenital diseases [88–90]. A recent visualization study of CSF circulation within the ventricular system of *Xenopus* [92, 93] and zebrafish larva [94, 95] indicated that these lower vertebrates are promising experimental models for further investigation of the early roles of CSF in body axis formation and brain development and several brain diseases associated with CSF circulation [92, 95, 96]. *Xenopus foxj1* was previously shown to play a significant role in MCC differentiation as well as ciliogenesis. Knockdown of *foxj1* using antisense morpholino oligonucleotides produces abnormal cilia and massively reduces CSF flow through the brain ventricles. The defective cilia cause blockage of CSF flow and hydrocephalus, which strengthens the idea that ciliary motility regulates CSF circulation [97]. Several other candidate genes play essential roles in ciliogenesis in *Xenopus*. Similar to *foxj1*, multicilin (downstream Notch signaling) is also essential for and sufficient to trigger MCC differentiation, and inhibition of multicilin causes severe defects in the MCC differentiation process [74]. *Cfap43* is a highly conserved gene in vertebrates that is abundantly expressed in MCC containing tissues including CP, and its functional protein is involved in the maturation of axoneme of motile cilia. Cfap43 mutant mice demonstrate dysfunctional motile cilia and hydrocephalus, whereas in *Xenopus*, cfap43 is a necessary for skin MCC and brain development [98]. Taken together, these studies indicate that motile cilia play a plausible role in CSF dynamics, and defects in motile cilia directly disturb CSF circulation. Thus, motile cilia play key regulatory roles in CSF movement in healthy brain.

### Hydrocephalus

Hydrocephalus is a brain disorder caused by blockage in CSF circulation, which causes accumulation of CSF in the brain ventricles and consequent ventriculomegaly. Typically, hydrocephalus can be divided into two groups. First, primary hydrocephalus (at birth), which is principally caused by genetic factors and developmental defects, frequently known as congenital hydrocephalus (CH) [99]. Second, acquired hydrocephalus (after birth), which may be attributable to several external factors, including brain injuries, hemorrhage, and pathogenic infections [99]. In CH, genetic factors can be divided into two subgroups. First, genes encoding products involved in cilia biogenesis/maturation. Second, genes encoding products entirely excluded from the process of cilia biogenesis/maturation (non-ciliated genes). These factors include cytokines, growth factors, and other components of signaling pathways with direct involvement in brain development and growth. Genes in the second category encode products exclusively involved in cilia formation and related signaling pathways (or cilia-based causes of hydrocephalus). In non-cilia related hydrocephalus, mutations in *L1CAM* (encoding L1 cell adhesion molecules, gene located at Xq28) are common in CH, generally referred to as L1 syndrome (*L1CAM*-associated hydrocephalus) or X-linked hydrocephalus [100–102]. Recently, whole-exome sequencing of samples from 381 CH patients revealed that a large portion of genes are involved in the regulation of neural stem cell biology, transcriptional mechanisms, and early neural development [103, 104]. Non-cilia related hydrocephalus is beyond the topic of this review, so we have focused on the first category of genes (cilia related causes of hydrocephalus). However, in acquired hydrocephalus, normal pressure hydrocephalus (NPH), a potentially curable, neurological syndrome, was first reported by Hakim and Adams [105] and its symptoms may include ventriculomegaly, gait apraxia, and urinary incontinence. However, the intracranial pressure in NPH remains normal, which is why it is termed NPH [105]. NPH is classified into idiopathic (iNPH) and secondary (sNPH) subtypes. Generally, iNPH is observed in adults (commonly older than 60 years), whereas sNPH can develop at any stage of life [106]. NPH has recently been reviewed by Oliveira et al. [106]. Additionally, post-hemorrhagic hydrocephalus (PHH) is another brain disorder typically caused by impaired CSF flow followed by hemorrhage in the brain [107]. The causes, intervention strategies, and potential therapeutic targets of PHH have been discussed by Chen et al. [108]. In motile cilia-related disorders, the dysfunction of motile cilia and the consequent excessive accumulation of ventricular CSF are the primary characteristics of hydrocephalus [109]. Ventriculomegaly may also cause compression of the brain parenchyma and lead to further loss of cerebral volume and other neuropathological changes [110]. Although the causes of hydrocephalus have remained unclear for a long time, a growing body of evidence collected from human and experimental animal studies has helped improve our understanding of it. Genetic factors and molecular mechanisms are directly involved in the process of ependymal differentiation and ciliogenesis, and failure of these crucial regulatory processes might lead to different types of developmental/maturation defects producing dysfunctional cilia, eventually leading to hydrocephalus. At present, more than 100 causative genes have been identified that are directly involved in hydrocephalus and other ciliopathies associated with primary cilia [77, 111].

**Fig. 3:**
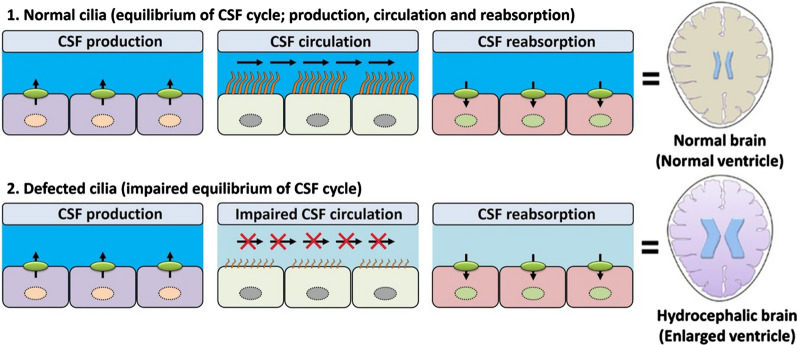
Effects of normal vs defective cilia on the CSF cycle (1). In normal cilia, the amount of CSF production, circulation, and reabsorption remain balanced. In balanced CSF equilibrium the brain is found in a healthy condition (2). In defective cilia, ciliary motility is severely impaired and leads to disturbed CSF circulation and equilibrium. The disturbed CSF circulation promotes ventriculomegaly and further hydrocephalus

Many neuropathological conditions can promote hydrocephalus in vertebrates. However, CH is thought to be the result of defects in cilia formation, improper CSF flow, and accumulation of CSF in the ventricular system during early brain development. Consistent with our theme, we have briefly reviewed the recent studies outlining the genetic causes of dysfunctional motile cilia and the development of life-threatening hydrocephalus. The axonemal protein of motile cilia, CCDC39, is also essential for the dynein motor protein regulatory complex, and loss-of-function mutations in *CCDC39* disturb the axonemal organization as well as ciliary beating [112]. *CCDC39* expression is precise and limited to CPs and ECs lining the forebrain of the embryonic mouse. The progressive hydrocephalus (prh) mutation in *CCDC39* (*CCDC39*^*prh/prh*^) causes severely impaired ciliary beating, which affects CSF movement and leads to the development of neonatal hydrocephalus in mice [113]. In contrast, appropriate EC differentiation and ciliogenesis is the result of several integrated signaling pathways, including PCP signaling, which is critical for proper positioning of exposed motile cilia by ECs. The Wnt-PCP signaling pathway plays a vital role in epithelial polarity and ciliary position. The cytoplasmic effector Dishevelled (Dvl), an intracellular adaptor of Frizzled (Fz), a Wnt receptor, is an essential agent that participates in both canonical and non-canonical Wnt pathways. The Dvl family includes the Dvl1, Dvl2, and Dvl3 proteins, and their encoding genes are commonly denoted as *Dishevelled* genes (*Dvls*). In mice, combined knockdown of *Dvls* results in normal EC differentiation but disrupted intercellular rotational alignment of ECs, which collectively result in hydrocephalus [114]. In addition, RSPH9 is a component of the radial spoke head complex, a thin stalk-like structure attached to the outer doublet microtubule of the motile cilia. The Rsph9^−/−^ knockout mice show disordered ciliary rotational beating, postnatal ventriculomegaly, severe sinusitis, and severe hydrocephalus [115]. A common observation in these conditions is that defective motile cilia result in significantly disrupted CSF circulation, which leads to ventricular dilation and severe neuropathological hydrocephalus. In the healthy brain, CSF dynamics are characterized by an equilibrium in CSF production (P), the amount of CSF in circulation (C), and the amount of CSF absorption (A). Stated simply, P = C = A = Healthy brain, while P ≠ C ≠ A = enlarged ventricles and a hydrocephalic brain (Fig. 3). If C is less than P, the additional uncirculated CSF will gradually accumulate in the ventricular system and increase the pressure on the ventricle wall and cause expansion. Eventually, the increasing pressure will start compressing the brain tissues such as the periventricular areas. Similar findings are reported in normal-pressure hydrocephalus, which further causes dementia [116]. Finally, we have reviewed the evidence regarding the different types of defective cilia phenotypes shown in Fig. 2 and their impact on the CSF turnover and causes of hydrocephalus (Table 1).

## Conclusions

The early development of the brain in vertebrates is characterized by many preprogrammed and decisive stages of differentiation, cell migration, and proliferation. Neurogenesis is initiated during gastrulation and proceeds until after birth. The gray and white matter volume begin to increase right after birth and the earliest form of CSF, known as eCSF, is found in the neural tube during neural tube growth [1–3, 117]. We have discussed the essential role of eCSF in early brain development, differentiation, and axis patterning. How does CSF influence the development and shaping of the mature architecture of the adult brain? In this review, we have characterized the continuous circulation of CSF within a healthy brain and the CSF-related functions. However, new findings with regard to the regulatory mechanisms of ciliogenesis have provided new insights and enhanced our understanding of how several classes of proteins come together to drive ciliary motility and other functions of mature motile cilia. These molecules integrate and interact with others in a precise, predefined, and spatiotemporal manner; even minor changes or deformations in any component of this complex machinery can destabilize these functional and physiological arrangements. The current challenges in understanding motile cilia can be categorized into questions regarding ciliary physiology and ciliary functions. The relationship between ciliary phenotypes and genotypes must be interpreted from a wide viewpoint; in this review we highlighted the five different types of ciliary phenotypes (Fig. 2 and Table 1), which collectively can show similar defects including impaired motility and defected CSF circulation. However, some ciliary factors have been demonstrated to have different physiological effects. For example, mutations in *DNAH17* (β-heavy chain of ODA) have been reported to be associated with poor sperm motility and male infertility, with no observed effects in respiratory cilia [118]. This indicates another future potential way to classify such organ/cell specific factors and to investigate their exclusive, context dependent, and compensatory physiological outcomes. Similarly, there distinct genetic mutations in *CEP290* (*centrosomal-cilia protein 290*) displayed in diverse syndromic ciliopathies [119]. Indeed, over 100 unique mutations (including non-sense, frameshift, and missense mutations) in distinct locations produce a variety of defective phenotypes (e.g., Senior Loken Syndrome and Mackel-Gruber Syndrome) (reviewed by Coppieters et al. [119]). Additionally, a mouse model of Cep290^gt/gt^ (produces a truncated protein) developed severe cystic kidneys and hydrocephalus. In contrast, Cep290^ko/ko^ (produces no protein) mice showed early vision loss and hydrocephalus [120]. These findings indicate differences in interaction or expression level between genes in a particular context (tissues/species) is dependent on the requirement of particular protein. However, future investigations are necessary in order to understand the complicated mechanisms or networks involved. Finally, the evidence presented here suggests that ciliary motility plays an important role in overall CSF circulation in vertebrates. We anticipate that this review might be helpful in advancing our understanding of the regulatory and functional roles of motile cilia in brain function and diseases.

## Data Availability

Not applicable.

## Abbreviations

BB: Basal body
CNS: Central nervous system
CP: Choroid plexus
CSF: Cerebrospinal fluid
CH: Congenital hydrocephalus
Dvl/Dsh: Dishevelled
EC: Ependymal cells
eCFS: Embryonic cerebrospinal fluid
MCC: Multiciliated cell
MTD: Microtubule doublets
ODA: Outer dynein arm
PCP: Planar cell polarity
prh: Progressive hydrocephalus
TF: Transcription factor
